# Hemisphere Tabulation Method: An Ingenious Approach for Pose Evaluation of Instruments Using the Electromagnetic-Based Stereo Imaging Method

**DOI:** 10.3390/mi14020446

**Published:** 2023-02-14

**Authors:** Zhongjie Long, Yongting Chi, Dejin Yang, Zhouxiang Jiang, Long Bai

**Affiliations:** 1 School of Electromechanical Engineering, Beijing Information Science & Technology University, Beijing 100192, China; 2 Key Laboratory of Modern Measurement & Control Technology, Ministry of Education, Beijing Information Science & Technology University, Beijing 100192, China; 3 Department of Orthopedics, Beijing Jishuitan Hospital, 4th Clinical College of Peking University, Beijing 100035, China

**Keywords:** pose evaluation, instrument localisation, stereo endoscope, 3D imaging, point cloud

## Abstract

Drilling of a bone surface often occurs in clinical orthopaedic surgery. The position and orientation of the instrument are the most important factors in this process. Theoretically, some mechanical components may assist in orienting an instrument to certain bone shapes, such as the knee joint and caput femoris. However, the mechanical assisting component does not seem to work in some confined spaces where the bone shape is a free-form surface. In this paper, we propose an ingenious hemisphere tabulation method (HTM) for assessing the pose accuracy of an instrument. The acquisition and assessment of HTM is conducted based on an electromagnetic-based stereo imaging method using a custom-made optical measurement unit, and the operation steps of HTM are described in detail. Experimental results based on 50 tests show that the HTM can identify ideal poses and the evaluated pose of an instrument location on a hemisphere model. The mean error of pose localisation is 7.24 deg, with a range of 1.35 to 15.84 and a standard of 3.66 deg, which is more accurate than our previous method.

## 1. Introduction

### 1.1. Background

**Figure 1:**
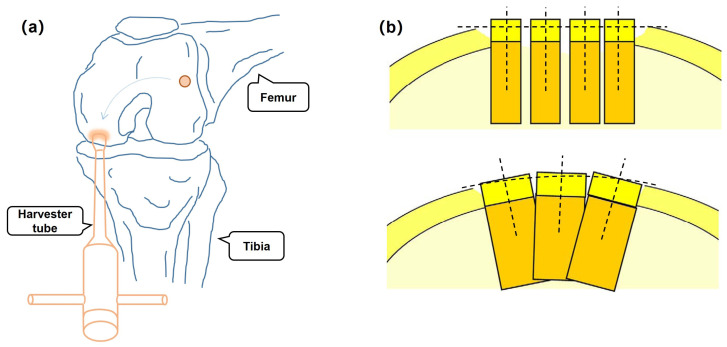
(**a**) Schematic representation of the OAT procedure. (**b**) Parallel graft insertion restores a flat surface, radial insertion can restore convexity [14].

Osteochondral lesions of the knee are very common in older people and athletic individuals, typically occurring as a result of osteochondritis dissecans or trauma. Individuals with this injury may suffer from swelling, pain, and early degenerative changes. Osteochondral autograft transplantation (OAT) is a reconstructive surgical procedure that addresses osteochondral lesions while maintaining the hyaline cartilage by replacing the defect area with a single osteochondral autograft [1]. As shown in Figure 1a, one or more cylindrical osteochondral plugs are harvested from a low-weight-bearing area of the femur and then injected into the defect region. However, the OAT procedure is not an easy task. Numerous studies [2,3,4] have suggested that both graft harvest and insertion must be performed perpendicular to the articular surface of the femoral condyle, as shown in Figure 1b. In other words, one of the crucial difficulties during OAT surgery is the optimal pose of the instrument, which is currently determined by the “feelings” of the surgeon.

### 1.2. Related Work

In this section, we present the related work in the context of pose estimation of tools that are employed in orthopaedic surgery. The potential of computer-assisted orthopaedic surgery to improve the accuracy and efficiency of surgery has been well proven [5]. Clues with regard to instrument poses or bone poses are displayed intra-operatively to surgeons to guide the manual task. Currently, the instrument/bone pose can be obtained by two ways: image-based methods and imageless methods. With image-based methods, the anatomical landmarks (e.g., implanted fiducials or surface points) are picked up by a surgeon with an optical tracked probe [6] and registered in a 3D model reconstructed from preoperative CT/MR images by surface rendering algorithms. In orthopaedic surgeries, however, it is hard to explicitly define this clearly, as the bone surface is not geometrical feature-rich and is usually exposed within a complex environment surrounded by blood and soft tissue. To this end, researchers have adopted neural networks to learn comprehensive semantics [7,8] using a certain amount of labelled datasets. A proof-of-concept study was outlined in [9], and a more systematic validation was reported in [10]. In imageless methods, the target bone is digitised with a tracked probe so that a generic kinematic and/or morphological model can be parameterised and adapted to it [11]. In addition, the resulting static pose needs to be further updated by dynamic target tracking (e.g., optical markers) because the bone inevitably moves during surgery. Hence, a dynamic reference frame containing infrared light-emitting diodes or infrared light-reflecting markers is pinned to the target bone. However, such procedures result in unnecessary incisions [12]. Recently, a novel markerless target tracking and registration was proposed in [13] to reduce such risks.

### 1.3. Motivation

Driven by the need for OAT, a pose evaluation approach that exploits information from a modified electromagnetic-based stereo imaging method (EBSIM) is proposed in this paper. The designed approach is ingenious and based on the statistical analysis of a transparent hemisphere model. On this basis, the instrument pose can be projected onto a medical treatment record paper by optical techniques. Although previous research on the pose evaluation and EBSIM concept has been published in [15,16], this paper is an improvement over the previously published works because of the following new additions:

1. A novel hemisphere tabulation method (HTM) for pose evaluation is briefly explained, which is different from the previous work and more accurate in pose assessment.
2. Experiments on a hemispheroid model are conducted by a shape measurement prototype to demonstrate the effectiveness of the proposed HTM.
3. The development of HTM provides an optics-based solution to OAT surgery in pose analysis of instruments, which can be seen as a baseline for accuracy comparison.

The rest of this paper is organised as follows. Section 2 introduces the preliminary works for stereo imaging, as well as the modified EBSIM and the proposed HTM. Section 3 shows the experimental results and analysis with the current method, followed by Section 4 which provides a discussion on the HTM. Finally, we give a brief conclusion in Section 5.

## 2. Methods

This section starts with an introduction of the imaging principle, a description of the design of the shape measurement unit, and a detailed description of the proposed HTM.

### 2.1. Principle of Imaging

**Figure 2:**
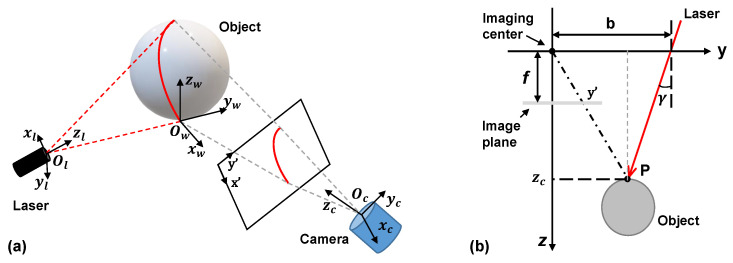
(**a**) Principle of the laser light section method. The laser beam falls on the object and is observed by the camera. (**b**) 3D coordinates of the laser light projected on the object surface can be reconstructed by the triangular principle. γ is the deviation angle with respect to the optical axis of the camera and *b* is the axial distance between the camera and the laser light source.

A laser light section method (LLSM) [15] depending on a pinhole model is applied to shape measurement, as shown in Figure 2a. Suppose a point P(x,y,z) in the world coordinate system is imaged on the camera sensor denoted as $p(x^{\prime },y^{\prime })$. The ideal pinhole camera’s imaging process can be expressed as

(1) $$z_{c}\left[\begin{array}{c} x^{\prime }\\ y^{\prime }\\ 1 \end{array}\right]=IP_{c}=\left[\begin{array}{ccc} f_{x} & 0 & c_{x}\\ 0 & f_{y} & c_{y}\\ 0 & 0 & 1 \end{array}\right]\left[\begin{array}{c} x_{c}\\ y_{c}\\ z_{c} \end{array}\right].$$

Here, I is the intrinsic matrix and $f_{x}$ and $f_{y}$ are the focal lengths in the *x* and *y* directions, respectively. $\left(c_{x},c_{y}\right)$ is the principal point coordinate and $P_{c}\left(x_{c},y_{c},z_{c}\right)$ is the point coordinate under the camera’s coordinate system.

Using Equation (1), we achieve the transformation from image coordinate to camera coordinate and obtain the expressions of $x_{c}$ and $y_{c}$, which have a linear relationship with respect to depth distance, $z_{c}$:

(2) $$x_{c}=\frac{z_{c}\left(x^{\prime }-c_{x}\right)}{f_{x}}$$

and

(3) $$y_{c}=\frac{z_{c}\left(y^{\prime }-c_{y}\right)}{f_{y}}.$$

In general, $f_{x}$ = $f_{y}$ = *f*, where *f* is the focal length of the camera. However, the depth distance, $z_{c}$, is still unknown in the pinhole model. Thus, we utilise a laser source to construct an optical model of the LLSM, as depicted in Figure 2b. On the basis of the trigonometric relationship yielded by the laser light, we obtain

(4) $$\frac{f}{z_{c}}=\frac{y^{\prime }}{b-z_{c}\cdot \tan\gamma }.$$

Thus, the depth distance can be expressed as follows:

(5) $$z_{c}=\frac{fb}{(y^{\prime }-c_{y})+f\cdot \tan\gamma }.$$

Using Equations (1) and (5), 3D coordinates on the object surface are then represented under the camera coordinate system by $P_{c}\left(x_{c},y_{c},z_{c}\right)$, in which parameters can be yielded from a classic camera calibration [17].

### 2.2. Development of a 3D Shape Measurement Unit Using the EBSIM

**Figure 3:**
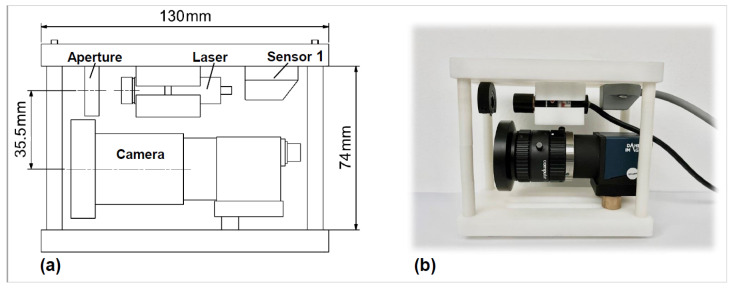
The developed 3D shape measurement unit. (**a**) Structural diagrams. The baseline between the camera and laser light source is 35.5 mm. (**b**) Side view of the prototype.

In our previous work, 3D shape measurements were reconstructed by an endoscope. However, a short baseline will decrease the measurement accuracy while utilizing the structural light technique. Thus, to demonstrate the effectiveness of the proposed HTM, a compact 3D shape measurement unit that has a long baseline was developed, as shown in Figure 3.

The measurement unit consists of four components. A commercially available camera (DAHENG-IMAGING, Beijing, China) with a resolution of 1280 × 960 pixels is adopted for the laser beam detection. A lens (Computar, Tokyo, Japan) with a 5 mm focal length is attached to the camera, obtaining the best capture distance of approximately 10 cm. In addition, an aperture is mounted on the front of the laser source (632 nm), so that the entire laser beam can be captured on the image plane. Meanwhile, such a design allows the user to choose the region of interest or scan smaller objects. Table 1 summarises the relevant parameters of the camera and lens. The camera is not equipped with a functionality to save previous frame data; therefore, an electromagnetic sensor is fixed inside the measurement unit for data acquisition using the EBSIM, which will be described in the next paragraph.

**Figure 4:**
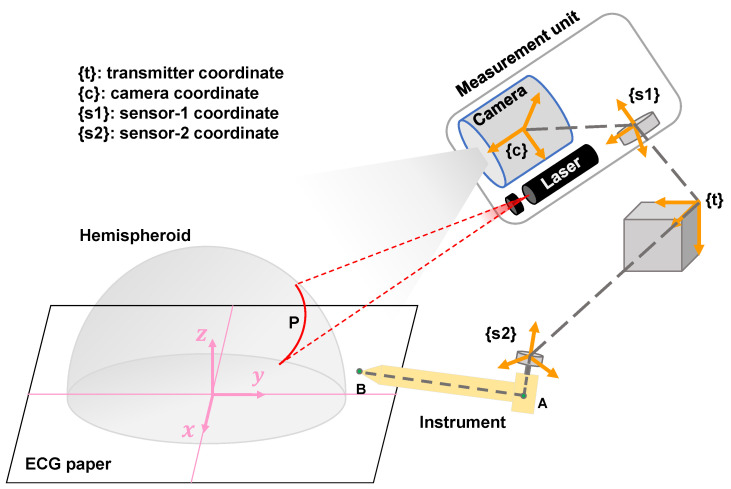
3D shape measurement and pose measurement using the EBSIM.

A state-of-the-art electromagnetic-based tracking system (LIBERTY, Polhemus, Colchester, VT, USA) was used to conduct 3D shape measurement and pose measurement using the EBSIM. The system consists of one transmitter and two sensors. The transmitter produces an electromagnetic field that acts as an accurate reference for position and orientation measurements for the sensors. Figure 4 illustrates the diagram of the coordinate transformation for 3D imaging.

We let $P_{s1}={\left[x_{s1},y_{s1},z_{s1}\right]}^{T}$ describe the same point $P_{c}$ with respect to the sensor 1 coordinate system as follows:

(6) $$\left[\begin{array}{c} P_{s1}\\ 1 \end{array}\right]=\left[\begin{array}{cc} {}_{c}^{s1}R & {}_{c}^{s1}T\\ 0 & 1 \end{array}\right]\left[\begin{array}{c} x_{c}\\ y_{c}\\ z_{c}\\ 1 \end{array}\right],$$

where ${}_{c}^{s1}R$ is the $\left(3\times 3\right)$ rotation matrix denoting the relative orientation of the camera with respect to the sensor 1 coordinate and ${}_{c}^{s1}T$ is the $\left(3\times 1\right)$ vector denoting the relative distance between sensor 1 and the camera.

In our case, we define the transmitter as the global coordinate of the tracking system. Both the measured 3D points and the instrument pose are constructed under the transmitter coordinate. Therefore, on the basis of Equation (6), the 3D coordinates of point $P_{c}$ with respect to the transmitter coordinate are finally represented as

(7) $$\left[\begin{array}{c} {}^{t}P_{s1}\\ 1 \end{array}\right]=\left[\begin{array}{cc} {}_{s1}^{t}R & {}_{s1}^{t}T\\ 0 & 1 \end{array}\right]\left[\begin{array}{c} P_{s1}\\ 1 \end{array}\right],$$

where ${}_{s1}^{t}T$ is the position vector from the transmitter to the sensor and ${}_{s1}^{t}R$ is the rotation matrix denoting the relative orientation of sensor 1 with respect to the transmitter centre, which is defined as follows:

(8) $$R_{\mathrm{ts}}=R_{\mathrm{ro}}\cdot R_{\mathrm{el}}\cdot R_{\mathrm{az}}.$$

Here, Euler angles, namely azimuth, elevation, and roll, represent an azimuth primary sequence of frame rotations (counter-clockwise rotation) that define the current orientation of the sensors corresponding to the zero-orientation state of the transmitter.

Similar to the 3D points, the instrument pose is computed and shown using the same global coordinate so that the scanned object surface can be navigated accurately. As depicted in Figure 4, the axial of the instrument pose is expressed by points *A* and *B*, which can be represented as

(9) $$\left[\begin{array}{c} {}^{t}P_{A}\\ 1 \end{array}\right]=\left[\begin{array}{cc} {}_{s2}^{t}R & {}_{s2}^{t}T\\ 0 & 1 \end{array}\right]\left[\begin{array}{c} d_{A}\\ 1 \end{array}\right]$$

and

(10) $$\left[\begin{array}{c} {}^{t}P_{B}\\ 1 \end{array}\right]=\left[\begin{array}{cc} {}_{s2}^{t}R & {}_{s2}^{t}T\\ 0 & 1 \end{array}\right]\left[\begin{array}{c} d_{A}+\mathrm{AB}\\ 1 \end{array}\right],$$

where $d_{A}$ is the position vector from sensor 2 to point A. Based on these two points, we can draw a line in the 3D space using the OpenGL technique, which represents the actual pose of the instrument.

### 2.3. Previous Pose Evaluation

**Figure 5:**
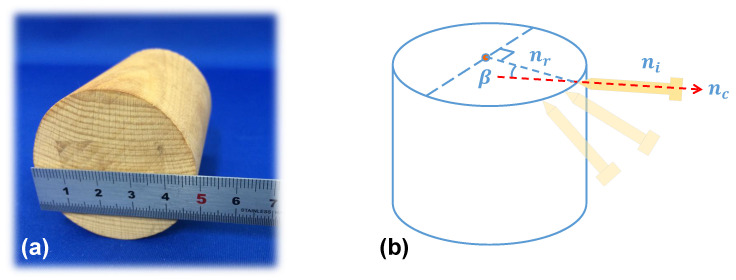
The previous method of pose evaluation. (**a**) Photos of the tested object. (**b**) Calculation of the angle difference.

Figure 5 shows our previous method of pose evaluation. A standard cylindrical piece of wood was used for 3D shape measurement and pose testing. A small area of the cylindrical surface was scanned, on which a number of points and its normal direction were marked. The central axis of the instrument, $n_{i}$, overlaps the normal direction, $n_{c}$, which is computed by a method based on geometric constraint solving [15] and considered the optimal angle/pose. However, the computed normal direction, $n_{c}$, is often biased by the errors in 3D shape measurement and the geometric constraint solving method. Therefore, the angle between $n_{c}$ and the real normal direction of the current position, $n_{r}$, is defined as the error angle β, as shown in Figure 5b.

One shortcoming of the previous evaluation method is that the real normal direction on the cylindrical surface is difficult to find and the error angle β is also hard to measure. Another shortcoming is that only the radial direction of $n_{i}$ is considered for pose adjustment, while the axial direction is missed. Thus, a modified pose evaluation method is needed to find the real errors.

### 2.4. The Proposed Hemisphere Tabulation Method (HTM)

To overcome the shortcomings of the previous method, an ingenious approach for pose evaluation of the instrument using an optics-based method is proposed. Four steps are required in the entire process and will be described in detail below: (1) Materials and Coordinate Definition, (2) Interest Point Marking, (3) 3D Shape Measurement, and (4) Pose Measurement and Calculation.

#### 2.4.1. Step 1: Materials and Coordinate Definition

**Figure 6:**
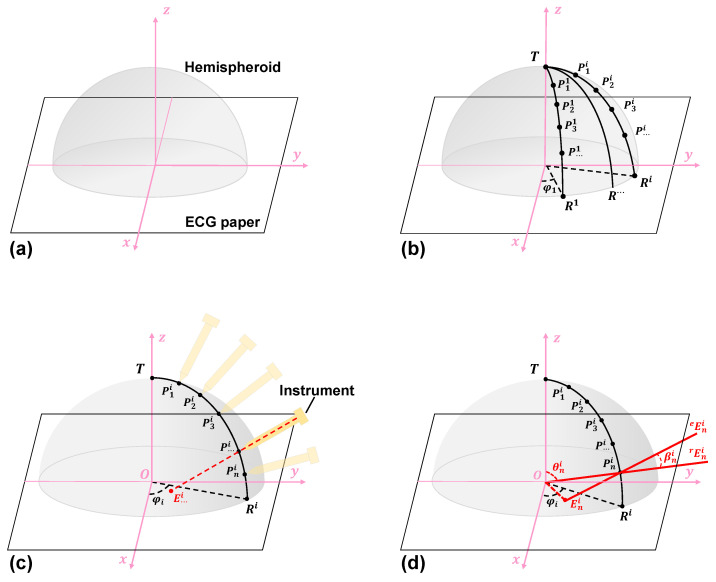
Processing steps of the HTM. (**a**) 3D coordinate definition on the ECG paper. (**b**) Arc marking on the shell. (**c**) Pose measurement on each interest point. (**d**) The computational principle of the pose evaluation.

To evaluate the pose, the first task is to describe the coordinates. Figure 6a shows the initial configuration which requires a hemispherical shell and ECG paper. First, we define the Cartesian coordinates, with the *x* axis pointing outward, the *y* axis pointing in the positive direction to the right of the ECG paper, and the *z* axis is perpendicular to the paper. We place a shell over the ECG paper, which is applied in the entire process, relating to the coordinate defined first. It is worth noting that the *z* axis passes through the vertex of the shell and the point is defined as T. Notably, a highlight of this method is that the shell is designed to be transparent; thus, the points from the instrument are recorded through an optical projection technique.

#### 2.4.2. Step 2: Interest Point Marking

Figure 6b shows the marking step. We first define the initial arc as $R^{1}$ and the initial angle as $\varphi_{1}$, also named the rotation angle, φ. $\varphi_{i}$, the corresponding angle of each arc, is a horizontal rotation angle with respect to direction and is defined as π/2i, where *i* is equal to the component. To have a high evaluation accuracy, the initial angle should be as small as possible (i.e., *i* as big as possible). The rotation angles in our system are equal, denoted as $\varphi_{1}=\varphi_{2}=\varphi_{\cdot \cdot \cdot }=\varphi_{i}$. Additionally, the intersection of the shell arc and the ECG paper is defined as $P^{i}$. Thus, arc $R^{1}$ is the line connecting point T and point $P^{1}$ on the shell. Starting with the *x* axis and rotating the arc projection around the *z* axis by $\varphi_{1}$, the initial arc $R^{1}$ is marked. Then, we mark *n* points on arc $R^{i}$ evenly, with the first point on the arc denoted as $P_{1}^{i}$, and the second, third, and so on as $P_{2}^{i}$, $P_{3}^{i}$..., $P_{n}^{i}$, respectively. After the above process, we rotate the shell around the *z* axis at an angle equal to φ, and mark a certain amount of arcs as $R^{2}$, $R^{3}$..., $R^{i}$, respectively. For simplicity, rotating the shell around the *z* axis is equivalent to the line connecting point T and point $P^{i}$ on the shell. Equivalently, just as we marked $R^{1}$ and $P_{n}^{1}$, we mark n points on the $R^{i}$ arc denoted as $P_{n}^{i}$.

#### 2.4.3. Step 3: 3D Shape Measurement

After the interest point marking is complete, the 3D shape reconstruction of the shell is conducted by the developed measurement unit. The experiment setup will be introduced in Section 3. The control circuit consists of a computer and an electromagnetic system. The measurement unit is controlled by the computer. Notably, the electromagnetic system, including a transmitter (source) and two sensors, extracts dates via the computer. The source generates a low-frequency magnetic field measured by the sensor. The source’s X-, Y-, and Z-axes are the default measurement reference frames. The sensor measures the low-frequency magnetic field generated by the source. The sensor is used to track both the position and orientation of the object to which it is attached relative to the measurement reference frame.

#### 2.4.4. Step 4: Pose Measurement and Calculation

Figure 6c shows a schematic diagram of the instrument pose evaluation. Actively, we select the arc $R^{i}$. Next, we place the instrument at certain points from $P_{1}^{i}$ to $P_{n}^{i}$ in order. A series of points on the other arcs are measured in the same way. The corresponding point on the shell will be projected onto ECG paper through the ray line. A ray line represents the component pose being projected onto point $E_{\cdot \cdot \cdot }^{i}$ on the ECG paper.

**Figure 7:**
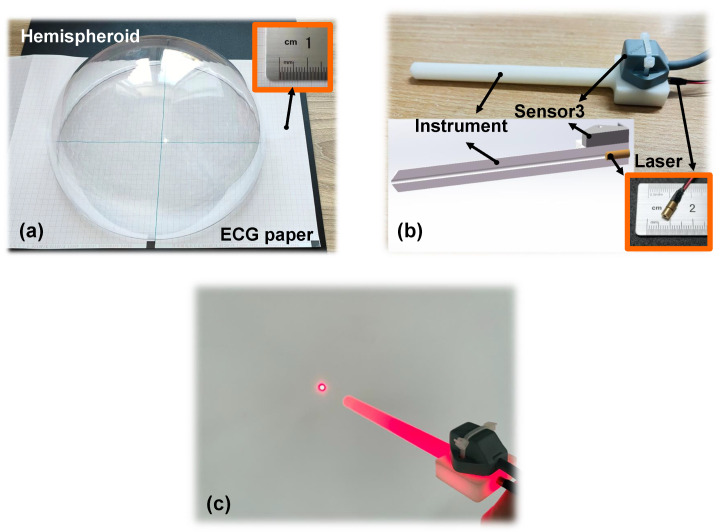
(**a**) Photograph of the hemispherical shell with ECG paper. (**b**) Custom-designed component that imitates an instrument. (**c**) Laser source passing through the instrument form a laser point.

Figure 7 shows the optics-based method and its component design. By implementing this method, we can measure the real normal vector on the shell surface. A hemispherical shell with a radius of 98.0 mm and recording paper (ECG paper) were used for pose evaluation and calculation, as shown in Figure 7a. A specific component that imitates a navigation instrument in the proposed method was designed using an acrylic board. As shown in Figure 7b, a point laser module was embedded at the top end of the component and its specifications were 3.8 mm × 13.8 mm, 1 mW, and 650 nm. In particular, the axis of laser light must be arranged to be coaxial with that of the component. In addition, an electromagnetic sensor 2, assisting in pose navigation, was mounted close to the top end of the component.

Figure 6d shows the computational principle of the pose evaluation. An arc $\overset{⌢}{TR^{i}}$, passing through the vertex of the shell centre, is evenly divided into *n* sections by (n−1) points, which are marked on the shell in advance. Based on the defined coordinate system, the 3D coordinates of the marked point (e.g., $P_{n}^{i}\left(x_{n}^{i},y_{n}^{i},z_{n}^{i}\right)$) can be expressed as follows:

(11) $$\left\{\begin{array}{c} x_{n}^{i}=r\sin\theta_{n}^{i}\cos\varphi_{i}\\ y_{n}^{i}=r\sin\theta_{n}^{i}\sin\varphi_{i}\\ z_{n}^{i}=r\cos\theta_{n}^{i} \end{array}\right.,$$

where *r* is the radius of the shell, $\theta_{n}^{i}$ is the corresponding angle of point $P_{n}^{i}$, and $\varphi_{i}$ is the horizontal rotation angle of $R^{i}$.

Meanwhile, the error angle, $\beta_{n}^{i}$, between the real, ${}_{}^{r}E_{n}^{i}$, and the estimated, ${}_{}^{e}E_{n}^{i}$, can be computed based on the law of cosines using the following equation:

(12) $$\cos\beta_{i}=\frac{{\bar{E_{n}^{i}P_{n}^{i}}}^{2}+r^{2}-{\bar{OP_{n}^{i}}}^{2}}{2r\bar{E_{n}^{i}P_{n}^{i}}},$$

where $\bar{E_{n}^{i}P_{n}^{i}}$ denotes the Euclidean distance of points $E_{n}^{i}$ to $P_{n}^{i}$ and $\bar{OP_{n}^{i}}$ denotes the Euclidean distance of point O to point $P_{n}^{i}$.

## 3. Experiments

### 3.1. Implementation Environment

The measurement system was implemented in a Windows10 20H2 environment using C++ (no GPU acceleration support) by three projects. The experiments, 3D shape acquisition and pose measurement, were both conducted in natural light using a laptop equipped with an Intel Core 2.7 GHz CPU, 8 GB memory, and one Intel HD 620 graphics card.

### 3.2. Results and Analysis

**Figure 8:**
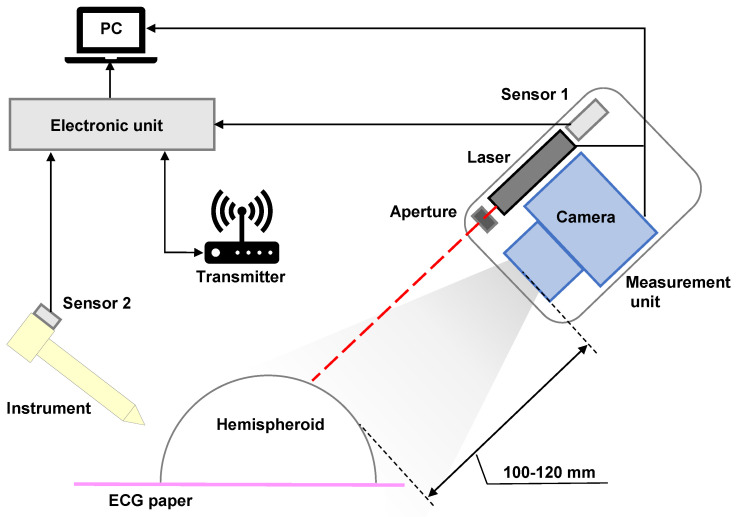
Experiment setup for 3D shape measurement and pose evaluation.

Pose evaluation using the proposed HTM was validated by a hemisphere shell. Figure 8 shows the experiment setup. A part of the shell was scanned freehand by the measurement unit with a distance of approximately 100 mm. Note that as the shell is transparent, imaging the laser beam seems to be difficult for the camera. Thus, the shell is painted by a coloured floating pen, and the colour can be easily removed when the shell is placed in water. In comparison, the interest points in the last step were marked by a non-erasable pen. Therefore, pose measurement on the shell will not be affected. All the point clouds were saved and displayed on the global coordinate according to Equation (7).

**Figure 9:**
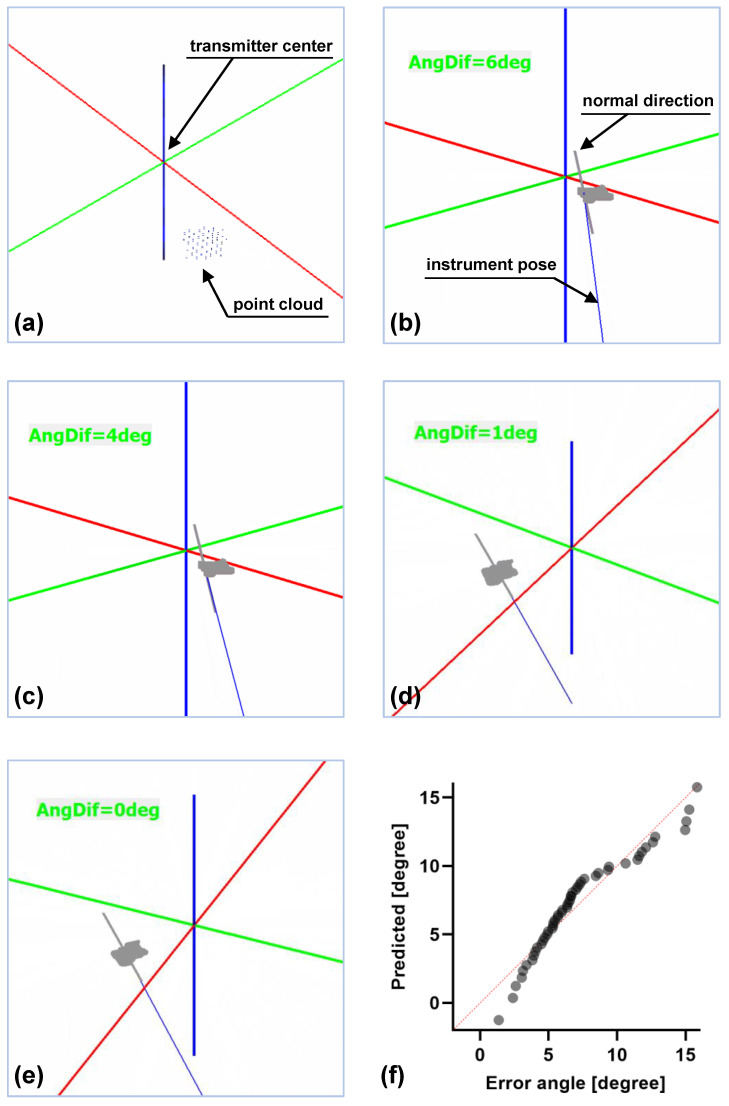
Experimental results on pose evaluation. (**a**) 3D point cloud (blue) of a partial shell. (**b**–**d**) Visual guidance displayed on a monitor to navigate the best pose. The instrument pose is adjusted. The error angles are 6 deg, 4 deg, and 1 deg. (**e**) The instrument pose (blue line) overlaps with the real normal direction (grey line) on the current position, with an optimal pose of zero error angle. (**f**) Results distribution of the error angle. Fifty positions are collected from the shell surface.

Figure 9a shows a view of the rearranged 3D point cloud under the transmitter coordinate system. Based on these points, the surface was then reconstructed in a few seconds using the marching cubes algorithm [18]. In accordance with the HTM steps, once the instrument tip touched the shell surface, a calculated normal direction was given for the current position and a line that represents the real-time pose of the instrument was displayed for the entire navigation process, as depicted in Figure 9b. Meanwhile, the localisation difference (i.e., the angle between the calculated normal direction and the pose of the instrument, not the error angle $\beta_{i}$) was computed and shown on the screen in real time to assist the operator in adjusting the instrument pose. For example, the error angles shown in Figure 9c–e are 4 deg, 1 deg, and 0 deg, respectively. In particular, 0 deg indicates the optimal pose for the instrument in the current position. Even so, the instrument pose is not the real normal direction in the current position. At this moment, we recorded the coordinates from the ECG paper, which are given by the projected laser light. Based on Equation (12), the error angle of the pose evaluation was finally obtained.

**Figure 10:**
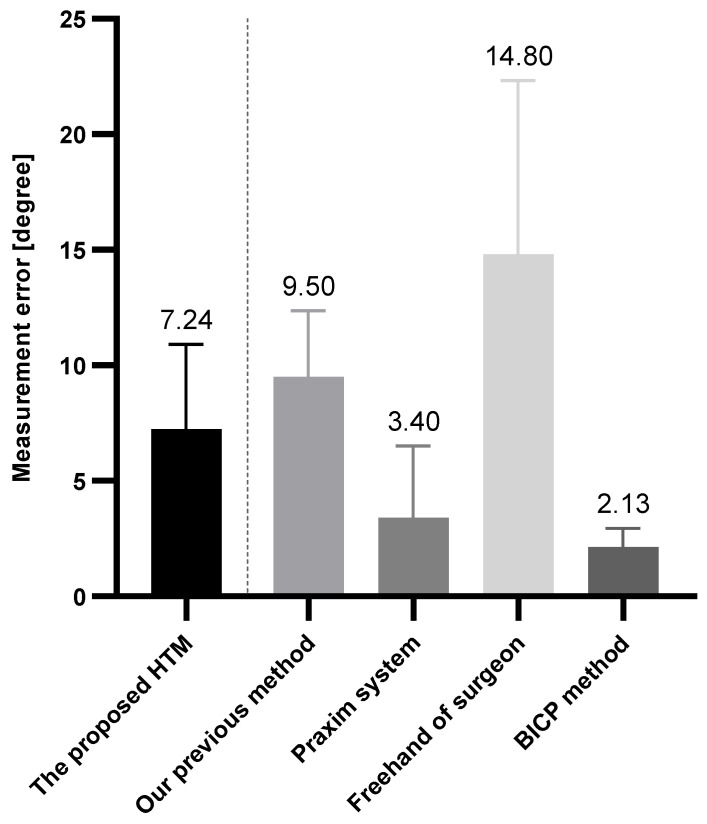
Comparison with localisation techniques and surgical navigation systems that were developed for OAT in the last ten years.

Figure 9f shows the normal distribution of the error angle results. This analysis is based on fifty position estimations of ten different model scans. For each scan, five estimations on different positions are measured. The experimental results show that the mean error of pose localisation is 7.24 deg, ranging from 1.35 to 15.84, with a standard deviation of 3.66 deg. Figure 10 depicts the comparison results of accuracy on instrument alignment. Note that the references we selected are within the field of orthopaedic knee surgery; other surgeries of body parts (e.g., total hip arthroplasty) are excluded. Compared to the existing navigation systems/techniques, the proposed HTM is more accurate than our previous method [15] and the freehand technique conducted by a professional surgeon [19]. However, the conventional image-free navigation system (Praxim, Grenoble, France) [19] and a recent BICP technique [20] are the state-of-the-art regarding the precision of instrument alignment of the OAT.

## 4. Discussion

Intraoperative instrument pose decision is a challenging task, especially in the orthopaedic scene/field where few reference substances are available due to the free-form bone surface. Acquiring the relevancy between the bone surface and the instrument is crucial for not only 3D reconstruction such as intraoperative measurement, but also enables the potential applications of skill evaluation [21]. We are able to achieve a promising pose evaluation result through our proposed EBSIM-based HTM (mean = 7.24 deg), which is more accurate than the previous method (mean = 9.5 deg) presented in [15]. An additional advantage arises from the fact the HTM allows placement of the hemispheric shell at any arbitrary orientation, offering operators the flexibility to choose the optimum placement of the shell that will not affect or restrict hand movements.

One of the limitations of our proposed method is that the HTM is developed based on a 3D representation obtained from the EBSIM; the object scan varies in material because of diffuse reflection, which directly affects the accuracy of the 3D shape measurement. Furthermore, pose estimation is constructed based on the geometric constraints of the 3D point cloud; therefore, a sparse cloud map will lead to a large error in pose evaluation. Another limitation originates from the inherent inaccuracy of the electromagnetic tracking system, which may be affected by external factors [22]. For example, the accuracy of the tracking system can be significantly reduced due to electromagnetic interference caused by metallic objects present in the operating field and in close proximity to the magnetic source. Therefore, to obtain accurate tracking results, the transmitter is usually mounted in a fixed position to a non-metallic stand, which is in close proximity to the sensors. In fact, the testing surface where the devices will be used (a table, for example) could have small metal hardware on it, such as scissors, screws, and bolts, which probably would not affect the accuracy of the tracking system. Hence, in our experiment, the transmitter is mounted on an aluminium tripod by an acrylic board. Nylon hardware such as plastic screws are only required when the hardware is in direct contact with the transmitter or sensors.

In the current study, an effort has been made to improve the shape measurement accuracy; a global shutter camera was selected to avoid fluctuation of the laser beam, while our the previous study used a rolling shutter. Although the accuracy and feasibility of the HTM are acceptable, we will continue to improve the rendered surface displayed in the graphical user interface, for example, by adding three forms to show the normal vector from the viewpoint of different axes.

## 5. Conclusions

This work contributes to existing clinical practices by introducing a pose accuracy evaluation of an instrument method that utilises stereo images obtained from a low-cost monocular measurement unit. The pose evaluation method was improved and validated by a transparent hemispheric model in which the normal direction of the current location can be known. The mean error in pose measurement was less than 8 deg compared to the actual pose. Furthermore, the proposed method does not require the employment of special calibration frames [23] or electromagnetic marker/sensor calibration [22], and it is easy to operate. Tested objects can be scanned from any position and orientation by the monocular measurement unit. In addition, the optical measurement unit can be replaced with a binocular stereo vision, which decreases the size compared to when using two mini cameras. In the near future, we hope that this work can be integrated into clinical endoscopes for pose decision support for the OAT procedure.

## Figures and Tables

**Table 1:** Specifications of the camera and lens.

Camera	Parameters of Camera	Lens	Parameters of Lens
Product Model	MER-133-54U3M/C	Product Model	Computar H0514-MP2
Sensor	Onsemi AR0135 CMOS	Focal length	5 mm
Scan mode	Global shutter	Control focus	Manual
Frame rate	54 frames per second	Focus range	10 cm–90 cm
Resolution	1280 (horizontal) × 960 (vertical) pixels	Angle of view	65.5 deg (horizontal) × 51.4 deg (vertical)
Dimensions	29 mm × 29 mm × 29 mm	Dimensions	Φ 44.5 mm × 45.5 mm

## Data Availability

Not applicable.
